# Opium trade and the spread of HIV in the Golden Crescent

**DOI:** 10.1186/s12954-017-0170-1

**Published:** 2017-07-21

**Authors:** Syeda Ayesha Farooq, Mohammad Hafiz Rasooly, Syed Hani Abidi, Kayvon Modjarrad, Syed Ali

**Affiliations:** 1 Aga Khan University, Karachi, Pakistan; 2 Afghanistan National Public Health Institute, Ministry of Health, Kabul, Afghanistan; 3 Nuffield Department of Clinical Medicine, University of Oxford, Oxford, UK; 4 US Military HIV Research Program, Walter Reed Army Institute of Research, Silver Spring, USA; 5 Department of Biomedical Sciences, Nazarbayev School of Medicine, Nazarbayev University, Astana, Kazakhstan; 6 Dow University of Health Sciences, Karachi, Pakistan

**Keywords:** HIV, Opium, Golden crescent, Afghanistan, Iran, Pakistan

## Abstract

The Golden Crescent region of South Asia—comprising Afghanistan, Iran, and Pakistan—is a principal global site for opium production and distribution. Over the past few decades, war, terrorism, and a shifting political landscape have facilitated an active heroin trade throughout the region. Protracted conflict has exacerbated already dire socio-economic conditions and political strife within the region and contributed to a consequent rise in opiate trafficking and addiction among the region’s inhabitants. The worsening epidemic of injection drug use has paralleled the rising incidence of HIV and other blood-borne infections in the region and drawn attention to the broader implications of the growing opiate trade in the Golden Crescent. The first step in addressing drug use is to recognize that it is not a character flaw but a form of mental illness, hence warranting humane treatment of drug users. It is also recommended that the governments of the Golden Crescent countries encourage substitution of opium with licit crops and raise awareness among the general public about the perils of opium use.

## Background

The Golden Crescent of South Asia comprises Afghanistan, Iran, and Pakistan [1, 2]. This region is considered a global hub for heroin and related opiate production and trafficking [3, 4]. In recent years, these countries have battled many challenges, including political turmoil, economic instability, war, and terrorism. The current review explores how such forces may have influenced the growth and expansion of the heroin trade within the region and how the trade has had implications for an epidemic of injection drug use, rising incidence of human immunodeficiency virus (HIV) and other blood-borne infections [5].

**Fig. 1:**
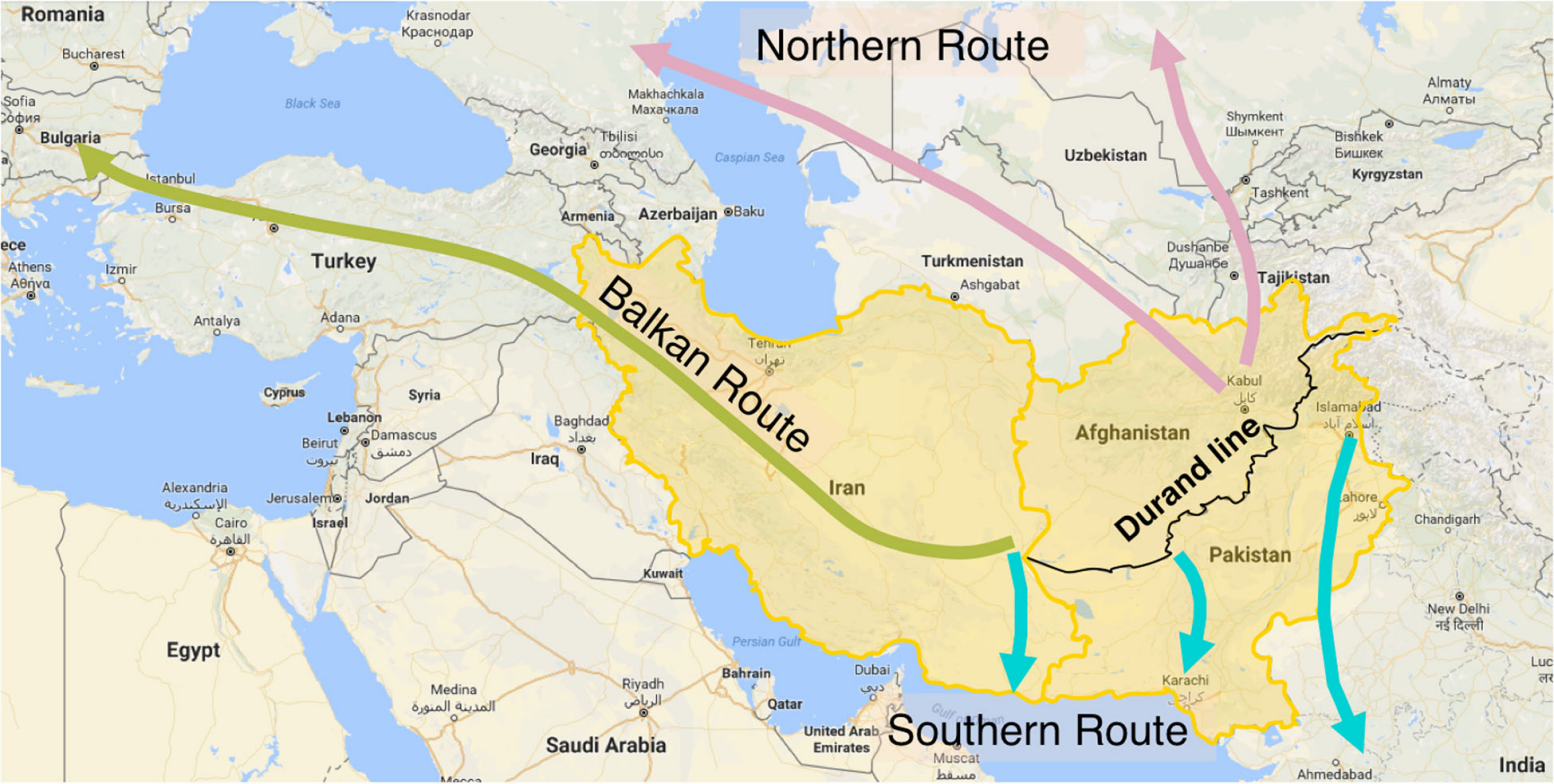
The Golden Crescent. The three Golden Crescent countries are indicated. *Pink*, *yellow*, and *blue arrows* indicate respectively the Northern, Balkan, and Southern routes of heroin trafficking [7, 104, 105]

There are three well-defined heroin trafficking routes that originate from the Golden Crescent region. The Balkan route operates through Iran and Turkey and traffics the bulk of Afghan heroin to Europe. The Northern route supplies heroin to the Russian Federation and Central Asia. Due to increased law enforcement along these two routes, alternate routes have emerged, collectively called the Southern route, that traffics heroin to Iran and Pakistan, and from these countries, via sea and air, to other parts of the world (Fig. 1) [6, 7].

Heroin is a derivative of opium that is obtained from the poppy plant, *Papaver somniferum* [8]. People addicted to heroin experience intense craving for the drug that interferes with their abilities to manage work, family, and other life commitments [9]. Long-term heroin use is thought to accelerate aging processes and is associated with liver and heart disease [10, 11]. Injecting heroin, through the sharing of contaminated needles, is also a major risk factor for blood-borne infections, such as HIV, hepatitis B virus (HBV), and hepatitis C virus (HCV). In a study of 483 people who inject drugs (PWID) recruited over a 2-year period from 2007 to 2009, Todd et al. found that those who had ever shared needles or syringes had higher odds of contracting HIV and/or HCV [5]. It is a common practice for PWID to—as a sign of brotherhood—share the same drug/blood-charged needle among themselves. A study conducted in Quetta and Lahore, Pakistan, found that approximately 92% of PWID practiced “jerking” where blood is drawn into the syringe and the drug/blood mixture is re-injected—a practice also referred to as “booting” or “registering” [12]. Some PWID also deliberately inject themselves with another’s blood in an effort to share the high or assuage the withdrawal symptoms—a practice referred to as “flashblood” or in Afghanistan, *khoon bozee* [5, 12].

Asia is home to two thirds of the world’s opiate users. At the end of 2006, the number of heroin users in Afghanistan, Iran, and Pakistan tallied 50,000 [13], 200,000 [14], and 484,000 [15], respectively. In 2009, these figures had risen to 120,000 [13], 391,000 [16], and 547,000 [16], respectively. Since the early 1990s, an HIV epidemic has been flourishing among PWID in Pakistan, where in 2011, one third of PWID were infected with HIV [17]. In Iran, a 2010 survey estimated the HIV prevalence among PWID to be 15% [18]. High prevalence of HIV, HBV, and HCV has also been reported among Afghan refugees [19]. In this article, we trace the heroin trade in the Golden Crescent region and postulate how it may have contributed to a rise in HIV and other blood-borne infection incidence, particularly among high-risk populations.

## Opium trade in Afghanistan

**Fig. 2:**
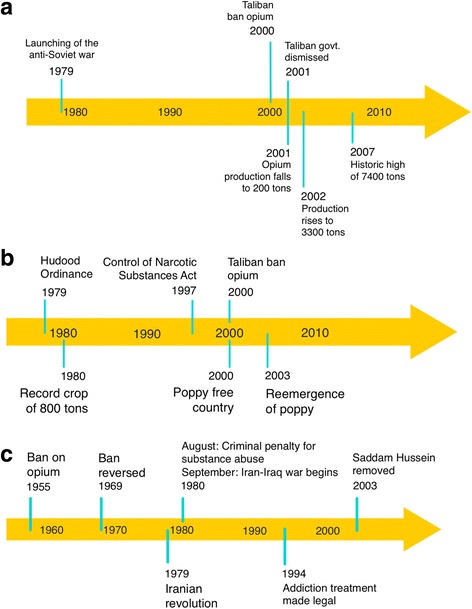
Timeline showing major events in the history of **a** Afghanistan [25, 26, 73, 106, 107], **b** Pakistan [54, 55], and **c** Iran [66, 67, 69, 70, 108] that directly affected the politics of the poppy in these countries

**Fig. 3:**
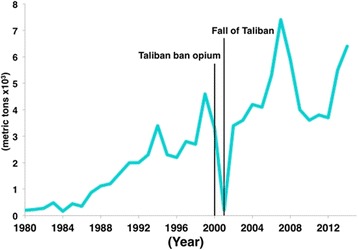
Opium production (metric tons) in Afghanistan from 1980 to 2014 [73, 106]

On Christmas Eve 1979, the army of the Soviet Union invaded Afghanistan. Over the course of the war’s 9 years, many *madrassas* (religious seminaries) materialized in Afghanistan [20] and Pakistan [21]. Many of the graduates of these *madrassas* formed the core of the Taliban movement that started besieging Afghanistan in 1993–1994 until the war with the USA in 2001 [22, 23]. The decade-long war with the Soviet Union had resulted in the destruction of the nation’s subsistence agriculture [24]. This, coupled with taxes paid to regional warlords, left farmers with little choice but to plant opium, a crop that requires little water and returns high profits. The profits obtained from the sale of opium were used to fund, among other things, the Afghan resistance to Soviet occupation. Opium production rose steadily throughout the 1980s and 1990s until 2000 when opium production fell precipitously on account of a *fatwa* issued by Taliban leader Mullah Omar that banned poppy cultivation [25, 26]. Opium production resumed after the fall of the Taliban in 2001 (Figs. 2a and 3). The opium trade, though not reliable, has been a relatively lucrative enterprise for Afghan farmers. Between 2002 and 2008, poppy farmers made $6.4 billion. The drug traffickers, on the other hand, earned $18 billion [7]. Much of the profits fell into the hands of warlords and Taliban militias, funding terrorist activities. As the opium trade funded terrorism in Afghanistan, it also weakened the integrity of civic institutions, further facilitating the illicit opium trade [27].

A 2010 study describes the following heroin use patterns in Afghanistan, based on patient profile: the war pattern, the refugees’ pattern, and the social and economic crisis pattern. The war pattern entails long-term drug use (>30 years) by ex-Afghan soldiers. The refugees’ pattern includes people who began to use drugs while they were refugees in Pakistan and Iran. The social and economic crisis pattern includes people who began to use drugs as a means of escape from their harsh realities [28]. The North Atlantic Treaty Organization (NATO) in 2008 declared that along with the war on terrorism, the war on drugs is also a prerogative of the international community and NATO troops can be employed to help with anti-narcotics operations [28].

## Injection drugs and HIV in Afghanistan

Three decades of conflict in Afghanistan have given rise to a shattered economy and disintegrated society. All available indicators suggest a rise in the incidence of depression and other mental illnesses [29]. Residents of a deprived nation looking for means of escape have increasingly turned to heroin, which has been cheap and plentiful in the country [30]. According to United Nations Office on Drugs and Crime (UNODC) estimates, 23,000 individuals injected drugs in Afghanistan in 2009, up from 19,000 in 2005. The Integrated Bio-Behavioral Survey (IBBS) conducted in Afghanistan in the same year showed that 27% of those who injected drugs also shared needles [31]. In 2012, the IBBS estimated the prevalence of HIV in those who inject drugs to be 4.4% [32, 33].

There is a real risk that HIV will spread from PWID to other high-risk populations with increasing frequency [30]. According to the IBBS 2012, the percentage of female sex workers (FSWs) living with HIV in Afghanistan was 0.3%. In 2009, there were more than 18,000 prisoners in Afghanistan, predicted to rise to 30,000 by 2015 [31]. Prisoners are considered to be at risk of contracting blood-borne infections because they are more likely to share needles, as they are scarce in prisons. Additionally, injection drug use is popular among prisoners, since injecting of drugs can be performed more surreptitiously than smoking. A study reports that those who inject in prison are five times more likely to be infected with HIV than those who do not. Prisoners may also face sexual abuse by prison guards or other prisoners, and this puts them at further risk for contracting HIV and other sexually transmitted infections (STIs) [34]. Lastly, men who have sex with men (MSM) are an underreported high-risk population in Afghanistan. The IBBS 2012 estimates the prevalence of HIV in MSMs to be 0.5%. From this same survey, it was found that two thirds of the MSMs in Kabul had unprotected anal intercourse in the previous year [31]. The most recent estimates place the prevalence of HIV in the general Afghan population at <0.1% [35]. In spite of the low prevalence, the rising incidence of HIV and other STIs among PWID, FSWs, MSMs, and prisoners [31, 36, 37] raise concerns that these infections might bridge into general populations.

## Afghan refugees in Iran and Pakistan

Afghan refugees constitute one of the biggest displaced populations in the world [38]. Following the 1978 communist takeover and the 1979 Soviet invasion, six million Afghans sought asylum outside their homeland, finding refuge all over the world, though primarily in Iran and Pakistan [39]. As of 2016, there were 1.5 million registered Afghan refugees in Pakistan and 951,000 in Iran [40]. The stresses of internal and external displacement of refugees has been shown to facilitate drug addiction and sex trade, putting them at risk for blood-borne and sexually transmitted infections [41–43]. A study comparing awareness and high-risk practices between Pakistani and Afghan drug users in Quetta, Pakistan, found that the Afghan refugees were more likely to practice injection drug use and needle sharing. The Afghans were less likely to know about HIV/AIDS or use a condom during sexual intercourse. Afghans were also less likely than Pakistanis to be educated or employed and more likely to be homeless [44]. It is not surprising that the prevalence of HIV and other blood-borne infections is high among Afghan refugees. In a sample of 556 refugees, one study found significant burden of HIV (6%), HBV (9%), and HCV (37%) [19].

In 2002, the United Nations High Commissioner for Refugees, under an agreement with the Afghan, Pakistani, and Iranian governments, began the largest repatriation program in history, aiming to send back 5.7 million Afghan refugees to their home country by 2012 [45]. By 2015, 5.8 million refugees had returned to Afghanistan [46]. While the program has been well intentioned from its outset, it has overlooked the implications of the high burden of infectious diseases in the repatriating population and the potential for resettling refugees to introduce novel strains of pathogens into the native Afghan communities [47].

In a study of Canadian Muslims, it was found that faith-based programs can be employed to combat the stigma associated with mental illness and addiction [48]. Cambodian refugees constitute the biggest refugee population in the USA, and a study conducted two decades after they first arrived here in 1975 found high rates of mental illness (depression and post-traumatic stress disorder (PTSD)) among the population. The study also found high rates (exceeding 90% in some categories) of exposure to trauma before migration, however, after arrival in the USA, the rates of exposure to trauma lessened [49]. A study assessed the prevalence of mental illness in the war-torn countries of Bosnia and Herzegovina, Croatia, Kosovo, Serbia, and Republic of Macedonia, among people who had been exposed to war between 5 and 15 years previously. In this population, the overall prevalence of mental illness was found to be 44.8%, with PTSD (10.6–35.5%) and major depression (4.1–37.3%) being the most frequent mental disorders [50]. Such studies highlight the need to promote long-term health for refugees and war affectees.

## Opiate production in Pakistan

**Fig. 4:**
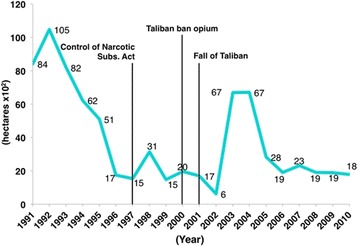
Opium cultivation (hectares) in Pakistan from 1992 to 2011 [71, 109, 110]

Since Pakistan gained its independence in 1947, its history has been interspersed with political upheaval and wars. In 1979, the Zia-ul-Haq government implemented the Hudood Ordinances, banning a set of what were deemed to be non-Islamic practices, among them being the production and use of intoxicating substances [51] (Fig. 2b). Ironically, that same year, Pakistan produced its record opium crop of 800 tons [52]. In the 1980s, Pakistan experienced a sharp increase in the number of heroin users: with virtually none reported in 1979 to 100,000 in 1983 [53]. This may have been a result of the anti-Soviet Afghan war that led to the flourishing of poppy fields at the Durand line that separates Pakistan and Afghanistan (Fig. 1). In 1997, the Control of the Narcotic Substances Act came about, and by the year 2000, Pakistan was declared a poppy-free country, meaning it had less than 1000 hectares under cultivation. Following the Taliban’s 2000 ban on opium cultivation in Afghanistan, prices rose and poppy fields re-emerged in Pakistan. In 2003, a record 6703 hectares were cultivated [54, 55]. In 2010, a 5-year Drug Control Master Plan was approved by the Pakistani parliament. This plan outlined strategies to curb drug manufacture and smuggling and to prevent and treat addiction [56]. (Figs. 2b and 4).

## Drug use and HIV in Pakistan

**Fig. 5:**
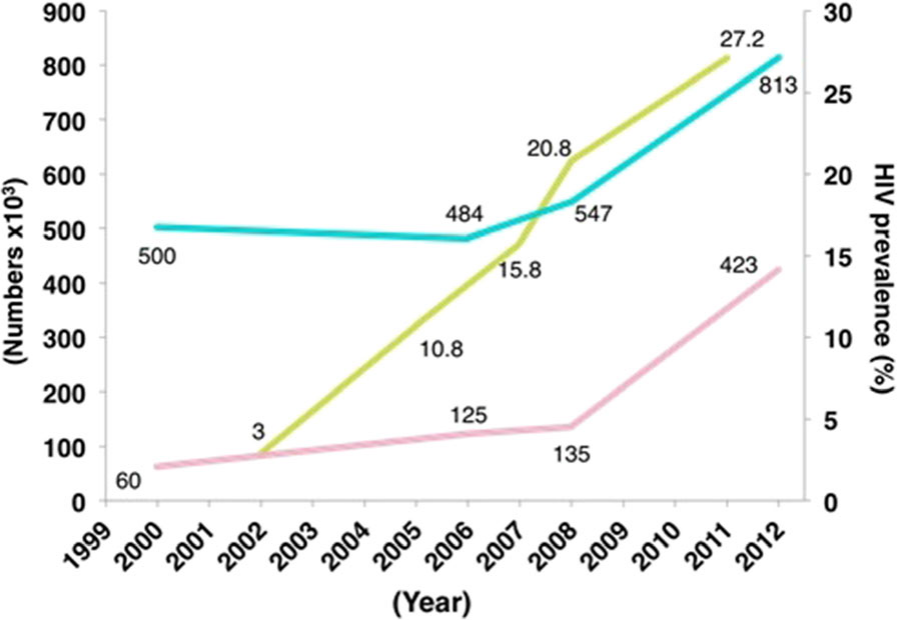
Heroin users, PWID, and the prevalence of HIV in PWID in Pakistan. HIV prevalence is shown from 2002 to 2011. Number of heroin users, PWID, and HIV prevalence is shown in, respectively, *blue*, *pink*, and *green* [15–17, 53, 111]

Although the overall HIV prevalence in Pakistan remains relatively low, it is alarmingly high in at-risk communities. In 2011, HIV prevalence among FSW, male sex workers (MSW), *Hijra* (transvestites) sex workers (HSW), MSM, and PWID was respectively 0.6, 1.6, 5.2, 7.2, and 27.2% [17, 57]. The earliest epidemic of HIV among PWID communities was thought to have been introduced by Pakistani migrant workers who were deported from the Gulf countries after having contracted HIV [58]. Evidence of a “founder effect” was shown in a PWID community infected with HIV subtype A, likely transmitted by a deportee from the Gulf [59, 60]. A survey conducted in the Pakistani city of Sargodha showed emergence of a new injecting behavior called “scale,” where an experienced street injector helps a novice, injecting almost half of a heroin mixture into the novice’s blood while saving the blood-contaminated rest of the mixture as payment or “scale” [61]. Figures from 2000 to 2011 show that there has been a steady rise in the population of PWID and heroin users in Pakistan. Not surprisingly, this rise closely aligns with an increase in HIV prevalence in this group from 3% in 2002 to 27.2% in 2011 (Fig. 5) [17].

Compared to PWID, the prevalence of HIV in other high-risk groups has stayed relatively low, albeit steadily rising, over the years. Mapping exercises have shown that there are 149,000 FSW in Pakistan. Data from 2011 show that 80% of FSW had heard of HIV/AIDS. Of these, most (94%) knew that HIV could be transmitted sexually, whereas only one third (33%) were aware that contaminated needles could be a source of transmission as well. A little more than 15% of the FSW reported that they had had intercourse with PWID, and 7% admitted to injecting drugs [17, 62]. HIV prevalence in the MSW population in Pakistan has increased from 0.4% in 2005 to 1.6% in 2011. While a majority (95%) of MSWs knew that HIV could be transmitted sexually, less than a half (46%) were aware that the virus could also spread through contaminated needles. In the same survey, 2% MSWs had injected drugs and 10% had sex with PWID [17, 62]. *Hijra* are a vulnerable, marginalized population; they are shunned by society and have limited access to healthcare services. According to 2014 estimates, approximately 50,000 HSW currently reside in Pakistan. According to a survey, although most (91%) HSW had good knowledge about HIV/AIDS, condom use with paying (24%) and non-paying partners (18%) was seriously lacking. Only 3% had injected drugs while 10% had had sex with PWID [17, 62].

Khanani et al. have reported a rising HIV epidemic in the MSM community in Pakistan. The study suggests that MSM who inject drugs may be responsible for bridging the epidemic between MSM and PWID [63].The MSM community in Pakistan is heavily stigmatized, since male-to-male sex is prohibited by religious edicts and social norms. MSM are therefore known to practice bisexuality while legally married to a female spouse. This hidden high-risk population acts as a vehicle for the transmission of HIV from the MSM population to the general population [58].

## Opiates in Iran

With 1.8 million opiate users in 2013, Iran has one of the highest opiate dependency rates in the world [64]. Social acceptance of opium smoking, in Iran, came about in the sixteenth century when it was first cultivated here [65]. During the 1920s, the opium trade constituted a quarter of Iran’s exports. In 1955, opium was banned in Iran, which may have indirectly contributed to the subsequent rise in opium production in Afghanistan and Pakistan. In 1969, the Iran reversed its policy and legalized opium cultivation [66], although drug trafficking remained a crime punishable by death (Fig. 2c) [67]. In June 1979, the Khomeini regime in Iran announced an International ban on currently illicit drugs, codifying it into law in January 1980. Drug use continued to increase, however, because neighboring Afghanistan produced large amounts of opium, which was trafficked into Iran, and westward from there. In September 1980, Iraq invaded Iran, giving rise to a conflict that lasted 8 years. As more and more resources were channeled into fighting the war, use of illicit drugs began to proliferate once again [68]. This war ended with a UN ceasefire in August 1988 [69]. After Saddam Hussein’s removal from Iraqi presidency in 2003, the Iran–Iraq border became porous, opening a new route for the trafficking of Afghan heroin [70].

According to a 1993 estimate, Iran had 3500 hectares of poppy under cultivation [71] much less than the hectarage noted in Pakistan (8185 ha.) [72] and Afghanistan (58,000 ha.) [73]. Follow-up surveys in 1998 and 1999 found no poppy being cultivated in Iran [64], whereas the 1999 figures for Pakistan and Afghanistan were respectively 1481 [72] and 91,000 ha. [73]. The reason for this rapid decline in poppy cultivation could be the competition—neighboring Afghanistan provided opium at much cheaper rates to the world market [71].

**Fig. 6:**
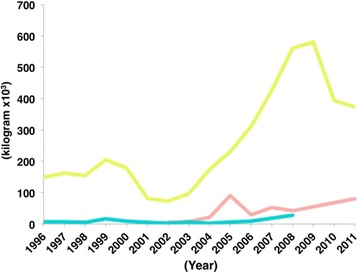
Opium seizures in Iran (*yellow*) [7, 78, 112, 113], Afghanistan (*pink*) [7, 16, 112], and Pakistan (*blue*) [7, 16] from 1996 to 2008

In a study of 1136 operating room patients, Alavi et al. found that 126 patients had used illicit drugs, with opium being the most frequently used drug (57%). The most common routes of drug use were swallowing (49.5%) and smoking (59%) [74]. In a national population size estimation study of illicit drug users, it was found that opium was the most common illicit drug used (1500 per 100,000 population), followed by shire (600), crystal methamphetamine (590), hashish (470), heroin/crack (350), methamphetamine/LSD/ecstasy (300), and injecting drugs (280). All types of substance use were more common among men than women, while people over 30 were more likely to inject drugs [75]. Iran has invested a billion dollars in border security to interrupt opium smuggling [76]. After establishing the Drug Control Headquarters in 1988, Iranian authorities intercepted increasing amounts of smuggled opium: from 21 metric tons of opium in 1990 [77] to 580 metric tons in 2009 [78] (Fig. 6). While the governments of Afghanistan and Pakistan have been concurrently seizing smuggled opium as well, the efforts by Iranian law enforcement authorities have been far more aggressive (Fig. 6). At the same time, the Iranian government was cracking down on illicit drug trade, a legal foundation was being laid for the treatment of people dependent on drugs without fear of prosecution or penalty of treatment seekers or providers. Consequently, the UNODC opened an office in Iran at the end of the 1990s and the first methadone clinic was opened in 2002 [70]. Iran is currently one of 22 countries that provides harm reduction services to prisoners [70]. However, a large number of prisoners in Iran are incarcerated on drug-related offences (between 1980 and 2000, as many as 1,700,000 were jailed on drug charges). In recognition of this fact, in 2005, Iranian judge Esmail Shooshtari ordered that drug users should be treated instead of punished [79, 80]. A systematic review of opioid use and treatment in the Persian Gulf states found that with the exception of Iran, harm reduction services were not available in that region due to the punitive Islamic laws that criminalize drug use. In all countries besides Iran, there was a scarcity of statistics on opioid use [81]. Harm reduction programs have been implemented successfully in other Muslim-majority countries such as Malaysia and have proved to be sustainable and cost-effective, preventing nearly 12,000 new HIV infections [82].

## HIV in Iran

There are three principal groups in Iran at high risk for HIV infection: PWID, FSW, and prisoners. In 2014, there were an estimated 170,000 to 230,000 PWID in the country [83]. The IBBS 2008 and 2010 reported an HIV prevalence of 15% among PWID [18]. In a qualitative study of 154 PWID living in Tehran, nearly two thirds were either unemployed or engaged in illegal jobs. Of these, 23 (15%) were women who reported engaging in sex work to fund their drug habit [84]. In 2010, there were an estimated 80,000 FSW in Iran. From the IBBS 2010, HIV prevalence among 872 FSW was estimated at 4.5%. Among these respondents, 74% had used drugs; of these, 21% admitted to injecting drugs. In their most recent sexual encounter, 57 and 36% FSWs reported condom use, with paying and non-paying clients, respectively [85]. According to IBBS 2009, HIV prevalence in Iranian prisoners was 2%. One fifth (21%) knew about HIV, and a quarter (25%) reported condom use in their last intercourse, whereas 17% had history of injection drug use and 13% had received tattoos in prison [86]. Another bio-behavioral survey conducted in 2012–2013 found the overall HIV prevalence among prisoners to be 1.4%. Among those who had been injecting drugs, it was 5% [83]. In a study of 252 prisoners in Iran, prevalence of HIV, HCV, and HBV was found to be, respectively, 15, 65, and 5%; 14% were found co-infected with HIV and HCV and 1% with HIV, HBV, and HCV [87]. There are no representative studies to gauge the HIV prevalence in the MSM population in Iran [83]. According to a 2011 report, 29% of unmarried, sexually active men in Iran have had anal sex with other men [88].

## Conclusion

The Golden Crescent continues to be plagued by an intransigent problem of opiate trade and use and a rising incidence and prevalence of HIV and other blood-borne infections. The governments in the three countries have made several efforts to address the issue of drug use as well as the rising HIV epidemics. Afghanistan, Iran, and Pakistan have ratified the three international conventions (1961, 1971, and 1988) that regulate the manufacture, trade, and use of illicit drugs [89]. Social acceptance of drug addiction as a disease and not a crime has been promoted in Pakistan through programs such as the organization Narcotics Anonymous that works under the Mary Adelaide Leprosy Center [90]. Such support groups are effective because they provide a resource for motivation and support for drug users [91]. In a similar vein, Iran has taken the step of making medical treatment for drug addiction legal [70] and opening clinics and support centers specifically for drug dependents [92]. Iran envisions legalizing opium and marijuana cultivation and seeks to abolish the death penalty for drug trafficking [93, 94]. In Afghanistan government and NGO-funded, harm reduction programs provide drug abstinence-based treatment. In 2002, there were only two treatment centers in Kabul, but by 2011, this figure rose to 14 [95]. The number of drug treatment centers across all of Afghanistan also rose from 43 in 2009 to 108 in 2013, with the capacity to treat more than 27,000 drug users [96]. The Organization for Harm Reduction in Afghanistan (OHRA) has been functional since 2011 and acts as an advocate for the vulnerable populations by screening, preventing, and treating HIV/AIDS [97].

Needle-exchange centers have been established in major cities of Pakistan to tackle needle-sharing practice among PWID. Needle-syringe exchange programs collect used syringes from the injectors and provide them with sterile ones. The workers at these centers also educate drug users about HIV and refer them to appropriate treatment centers. Studies have shown that HIV prevalence decreases in areas where such programs operate [98]. Such programs are the need of the hour and must be promoted in all three countries of the Golden Crescent.

Opium production may be curtailed in the Golden Crescent countries through different approaches. Financial incentives may be offered to encourage the farmers to cultivate alternative crops, such as saffron, pomegranate, grapes, and almonds [99, 100], that may bring financial returns comparable to revenues generated from poppy cultivation. Farmers can be convinced that such crops will provide a more steady income compared to opium, particularly as opium may be seized and destroyed by the law enforcement agencies. Governments should assist farmers in doing so by providing better irrigation, disseminating education about agriculture, distributing seeds, and setting up agriculture markets [100]. It has been proposed that the Afghan government should halt eradication of low-income farmers’ crops and instead focus on the wealthy landowners as they have more to lose. In fact, there is evidence that after eradication, wealthy landowners are less likely to replant opium as losing an opium crop can be costly and they would rather invest in licit, low-risk crops. Efforts need to be made to interdict drug traffickers. With international support, opium cultivation can be legalized in Afghanistan, as there is an unmet need in developing countries for opium-based medicines. Following the model of Turkey, Afghanistan could potentially step in to meet the licit opiate needs of the developing countries; however, it is challenging to legalize a crop such as opium as it requires political stability and expertise to prevent misuse [100]. Awareness should be engendered among the farmers on how opium can be harmful on a larger scale, facilitating the spread of infections and prostitution [101]. A recent publication from Pakistan shows how media can be effective in preparing a nation to think about matters related to sex and sexually transmitted diseases [102]. Another alternative to opium is the oil mining industry that could help kick-start the Afghan economy [103]. Public health experts, religious leaders, politicians, and the media need to engage in dialogue and be encouraged to collaborate in changing the mindset of poppy farmers, drug traders and users, commercial sex workers, and the general public about the menace of heroin use and of the growing HIV epidemic in the Golden Crescent region.

## Abbreviations

FSWs: Female sex workers
HBV: Hepatitis B virus
HCV: Hepatitis C virus
HIV: Human immunodeficiency virus
HIV/AIDS: Human immunodeficiency virus/acquired immunodeficiency syndrome
HSW: *Hijra* sex workers
IBBS: Integrated Bio-Behavioral Survey
MSM: Men who have sex with men
MSW: Male sex workers
OHRA: Organization for Harm Reduction in Afghanistan
PWID: People who inject drugs
STI: Sexually transmitted infections
UNHCR: United Nations High Commissioner for Refugees
UNODC: United Nations Office on Drugs and Crime
